# Association of depressive disorders and dementia with mortality among older people with hip fracture

**DOI:** 10.1186/s12877-023-03862-w

**Published:** 2023-03-09

**Authors:** Erika Olofsson, Yngve Gustafson, Sebastian Mukka, Eva Tengman, Lenita Lindgren, Birgitta Olofsson

**Affiliations:** 1 Department of Nursing, Umeå University, Umeå, Sweden; 2 Department of Community Medicine and Rehabilitation, Geriatric Medicine Division, Umeå University, Umeå, Sweden; 3 Department of Surgical and Perioperative Sciences, Umeå University, Umeå, Sweden; 4 Department of Community Medicine and Rehabilitation, Physiotherapy Division, Umeå University, Umeå, Sweden

**Keywords:** Dementia, Depressive disorders, Hip fracture, Mortality, Older people

## Abstract

**Background:**

Hip fracture (HF) is a significant cause of mortality among older people. Almost half of the patients with HF have dementia, which increases the mortality risk further. Cognitive impairment is associated with depressive disorders (DDs) and both dementia and DDs are independent risk factors for poor outcome after HF. However, most studies that evaluate mortality risk after HF separate these conditions.

**Aims:**

To investigate whether dementia with depressive disorders (DDwD) affects the mortality risk at 12, 24, and 36 months after HF among older people.

**Methods:**

Patients with acute HF (*n* = 404) were included in this retrospective analysis of two randomized controlled trials performed in orthopedic and geriatric departments. Depressive symptoms were assessed using the Geriatric Depression Scale and cognitive function was assessed using the Mini-Mental State Examination. A consultant geriatrician made final depressive disorder and dementia diagnoses using the Diagnostic and Statistical Manual of Mental Disorders criteria, with support from assessments and medical records. The 12-, 24- and 36-month mortality after HF was analyzed using logistic regression models adjusted for covariates.

**Results:**

In analyses adjusted for age, sex, comorbidity, pre-fracture walking ability, and fracture type, patients with DDwD had increased mortality risks at 12 [odds ratio (OR) 4.67, 95% confidence interval (CI) 1.75–12.51], 24 (OR 3.61, 95% CI 1.71–7.60), and 36 (OR 4.53, 95% CI 2.24–9.14) months. Similar results were obtained for patients with dementia, but not depressive disorders, alone.

**Conclusion:**

DDwD is an important risk factor for increased mortality at 12, 24, and 36 months after HF among older people. Routinely assessments after HF for cognitive- and depressive disorders could identify patients at risk for increased mortality, and enable early interventions.

**Trial registration:**

RCT2: International Standard Randomized Controlled Trial Number Register, trial registration number: ISRCTN15738119.

## Introduction

The hip fracture (HF) incidence increases with age, and HF is a significant cause of mortality among older (aged < 60 years) people [1, 2]. The mortality risk increases shortly after HF, and persists for 10 years [3, 4]; predictors are high American Society of Anesthesiologists scores, pre-fracture mobility, and dementia [5, 6].

Almost half of patients sustaining HFs have dementia [6–8] and increased short- and long-term mortality [9, 10]. Cognitive impairment/dementia is associated with depressive disorders (DDs) [11, 12], which are more common among patients sustaining HF than in the general population [13, 14]. Older patients with DDs have an increased fall risk [15] and greater comorbidity burden [16]. Patients with DDs, in addition to comorbidity, have an increased 1-year mortality risk [17]. However, knowledge of DDs’ effect on mortality among patients sustaining HFs is limited.

DDs and dementia are independent predictors of poor outcomes and increased mortality risk after HF [18]. However, most studies that evaluate mortality risk after HF have separated these conditions, and to our knowledge, only one small study, has explored whether dementia with depressive disorders (DDwD) affects long-term mortality after HF surgery, which revealed a significantly increased 12-month mortality risk [19]. The present study has the aim to investigate whether DDwD affects the mortality risk at 12, 24, and 36 months after HF among older people.

## Method

### Study design and participants

Data used in this secondary analysis of 404 patients with acute HFs were from two randomized controlled trials (RCT1 and RCT2). Both RCT were performed in the orthopedic- and the geriatric departments of Umeå University Hospital, Sweden. RCT1 included 199 patients with femoral-neck fractures enrolled in May 2000-December 2002 [20]; RCT2 included 205 patients with femoral-neck and trochanteric-region fractures enrolled in May 2008-June 2011 [21]. Inclusion criteria for both studies were age ≥ 70 years and Umeå municipality residence. Exclusion criteria for RCT1 were severe rheumatoid arthritis/hip osteoarthritis, pathological/hospital-acquired HF, renal failure, and bedridden before the fracture occurred. Those for RCT2 were hospital-acquired/pathological HF. Both RCTs included patients with cognitive impairment or dementia.

### Randomized controlled trials

Participants in both RCTs were randomized consecutively to intervention and control groups. In RCT1, participants in the intervention group received postoperative care according to a multifactorial rehabilitation program in a specialist orthopedic geriatric unit. Multidisciplinary teams performed comprehensive geriatric assessment, management, and rehabilitation to prevent, detect and treat early postoperative complications. Control participants received conventional postoperative care in a specialist orthopedic [20].

In RCT2, all patients received postoperative care in the geriatric orthopedic department according to the multifactorial rehabilitation program (now the standard of care, based on RCT1 results). The intervention focus was early discharge, with rehabilitation in patients’ homes supported by an interdisciplinary geriatrics team [21]. As no difference in survival was detected in either RCT, data from all participants were analyzed together.

Randomization, recruitment, and intervention contents have been described previously [20, 21]. Participants in both RCTs received written and oral information, and they or their next of kin provided consent. Participants and/or next of kin were informed that they could withdraw at any time with no repercussion. The RCTs were approved by the Regional Ethical Review Board in Umeå, Sweden (Dnr 00–137 and Dnr 08-053 M), and amendments for this study was approved by the Swedish Ethical Review Authority (Dnr 2021–00,024 and Dnr 2021–00,681). All methods were performed in accordance to the Declaration of Helsinki.

### Data collection

At baseline, research assistants (nurses, physiotherapists, and occupational therapists) collected medical, functional, and social data from the patients, their relatives, and/or medical charts. In RCT1 and RCT 2, assessments were performed at 3–5 days postoperatively. Dates of death were collected from medical records.

Participants’ pre-fracture indoor walking ability was assessed [scale, 1 (no functional ability/need for two people’s assistance)–7 (normal function)] [22]. Walking device use was registered. Pre-fracture independence in personal and instrumental activity of daily living (ADL) was assessed using the Katz ADL index [23]. Patients’ vision (ability to read 5-mm block letters with/without glasses) and hearing (ability to hear normal speech with/without hearing aids for 1 m) were assessed.

### Outcome measurements

The outcome was 12-, 24- and 36-month mortality following HF surgery. DDs diagnoses were based on current antidepressant treatment and Geriatric Depression Scale (GDS-15) scores (scores ≥ 5 indicate significant depressive symptoms) [24]. In addition, fluctuations of clinical state were observed and registered by the Organic Brain Syndrome scale (OBS scale) [25]. The GDS-15 has good sensitivity and specificity, even among very old people with low Mini Mental State Examination (MMSE) scores, for DDs detection according to the Diagnostic and Statistical Manual of Mental Disorders – Text revision (4.^th^ ed.; DSM-IV-TR) [26, 27]. Cognitive function was assessed using the MMSE, (scores 0–30, scores ≤ 23 indicate significant cognitive impairment) [28].

At the end of the RCTs, a consultant geriatrician (YG), blinded to group allocation and not employed at the wards, set all diagnoses. The consultant geriatrician used all possible information from patient’s medical record (diagnoses, complications, and other important documentations), patient’s prescribed drugs and assessments performed in these RCTs (including the MMSE, GDS- 15, Philadelphia Geriatric Center Morale Scale (PGCMS) [29, 30], Katz ADL index, OBS Scale), as well as vision and hearing tests, to determine whether participants fulfilled the DMS-IV-TR criteria for DDs and dementia.

### Statistics

Baseline characteristics were compared between surviving and deceased participants at 36 months after HF. Pearson’s chi-square test and Fisher’s exact test were used to analyze categorical variables. Normally and non-normally distributed data were compared between groups using the independent-sample t and Mann–Whitney U test, respectively.

To identify independent factors predicting 36-month post-HF mortality; correlations between baseline variables were tested using Pearson’s and Spearman’s coefficients (< 0.6 for all covariates) [31]. Univariate analysis was performed to examine unadjusted associations of potential risk factors with HF and increased mortality risk. Variables with *p* ≤ 0.10 (*t* or chi-squared test) were subjected to multivariable logistic regression using the forward stepwise conditional method [32]. Odds ratios (ORs) with 95% confidence intervals (CIs) were calculated to identify factors associated with 12-, 24-, and 36-month mortality. Adjusted analysis was used to obtain ORs for depressive disorders with dementia (DDwD).

Statistical analyses were conducted with SPSS (version 25; IBM Corporation, Armonk, NY, USA). *P* < 0.05 was considered to indicate significance.

## Results

### Patients and descriptive

The sample comprised 404 patients [295 (73.0%) women, mean age 82.6 ± 6.5 years]. Most (87.5%) participants walked independently before HF. More participants who died than survived within 36 months had the comorbidities examined. Vision and hearing impairment frequencies did not differ significantly between groups. At baseline, 153 (38.5%) patients had DDs and 167 (41.3%) had dementia; 94 (23.3%) patients had DDwD. Fifty-nine (15.0%) had DDs without dementia and 72 (18.0%) had dementia without DDs. One patient with dementia could not perform assessment for DDs, and is missing (Table 1). DDs correlated weakly to moderately with dementia (*r* = 0.31).

**Table 1:** Study population characteristics and comparison of the 404 patients deceased or survived within 36 months after hip fracture. Continuous variables are presented with mean and standard deviation

	Total	Deceased	Survivors	*P*-value
	No. 404	36 months No. 168	36 months No. 236	
Age	82.6 (± 6.5)	83.8 (± 6.5)	81.7 (± 6.4)	**0.002**
Sex				
Female	295 (73.0%)	113 (67.3%)	182 (77.1%)	**0.037**
Living in nursing home	136 (33.7%)	76 (55.9%)	60 (44.1%)	**< 0.001**
Not having a partner	293 (72.5%)	129 (76.8%)	164 (69.5%)	0.132
Independent in walking indoors before fracture	350 (87.5%)	135 (82.3%)	215 (91.1%)	**0.014**
Independent in P-ADL before fracture	173 (43.3%)	40 (24.4%)	133 (56.4%)	**< 0.001**
Normal vision	231 (61.8%)	90 (60.4%)	141 (62.7%)	0.740
Normal hearing	299 (76.5%)	119 (73.9%)	180 (78.3%)	0.381
Pulmonary disease	58 (14.5%)	34 (20.7%)	24 (10.3%)	**0.006**
Cerebrovascular disease	94 (23.5%)	48 (28.9%)	45 (19.7%)	**0.042**
Cardiovascular disease	215 (53.9%)	108 (65.1%)	107 (45.9%)	**< 0.001**
Cancer (current)	41 (10.3%)	29 (17.7%)	12 (5.1%)	**< 0.001**
Diabetes	72 (17.9%)	38 (22.8%)	34 (14.5%)	**0.045**
Depression pre fracture	154 (38.5%)	83 (50.3%)	71 (30.2%)	**< 0.001**
Depression, but not dementia	60 (15.0%)	24 (14.5%)	36 (15.3%)	0.943
Dementia pre fracture^a^	167 (41.3%)	94 (56.0%)	73 (30.9%)	**< 0.001**
Dementia, but not depression	72 (18.0%)	34 (20.6%)	38 (16.2%)	0.315
Dementia and depression	94 (23.5%)	59 (35.8%)	35 (14.9%)	**< 0.001**
Fermoral neck fracture	329 (81.6%)	140 (83.3%)	189 (80.4%)	0.540
Undisplaced/minimally displaced (B1)	88 (21.8%)	40 (45.5%)	48 (54.5%)	
Basicervical (B2)	14 (3.5%)	10(71.4%)	4 (28.6%)	
Displaced (B3)	226 (56.3%)	90 (39.6%)	137 (60.4%)	
Intertrochanteric fracture	74 (18.4%)	28 (16.7%)	46 (19.6%	
Trochanteric (A1-A2.1)	38 (9.4%)	11 (28.9%)	27 (71.1%)	
Trochanteric (A2.2-A2.3)	21 (5.2%)	10 (47.6%)	11 (52.5%)	
Trochanteric (A3)	15 (3.7%)	7 (46.7%)	8 (53.3%)	

^a^Diagnostic assessment for depressive disorder was not performed in one patient with dementia

*P-ADL* Personal activities of daily living

### Mortality

The overall 36-month mortality rate was 41.6%; it was significantly higher among older patients, nursing home residents, and patients with pre-fracture personal ADL dependence. Significant differences between deceased and surviving participants were observed for the comorbidities examined (Table 1). Mortality was equivalent between participants in RCT1 and RCT2, and between intervention and control groups (data not shown).

Half of the patients who had died at 36 months had DDs (50.3%) or dementia (56.0%) preoperatively. Fifty-nine (35.8%) patients with DDwD,14.5% of those with DDs alone, and 20.6% of those with dementia alone died within 36 months.

The mortality risk among patients with dementia alone was increased at 12 (OR 4.05, 95% CI 1.52–10.79), 24 (OR 2.87, 95% CI 1.38–5.98), and 36 (OR 2.55, 95% CI 1.30–4.99) months. No significant increase was observed for those with DDs alone. Patients with DDwD had increased mortality risk at 12 (OR 4.67, 95% CI 1.75–12.51), 24 (OR 3.61, 95% CI 1.71–7.60), and 36 (OR 4.53, 95% CI 2.24–9.14) months (Table 2).

**Table 2:** Multivariable logistic regression of mortality risk at 12-, 24- and 36 months after a hip fracture with the combination of depressive disorders and dementia

Variable	12 months		24 months		36 months	
	OR	CI	OR	CI	OR	CI
Age	**1.08**	1.03–1.14*	**1.07**	1.02–1.10*	**1.05**	1.01–1.09*
Sex						
Female	1.0	ref	1.0	ref	1.0	ref
Male	1.29	0.63–2.62	1.70	0.98–2.96	1.33	0.78–2-26
Resident						
Own home	1.0	ref	1.0	ref	1.0	ref
Nursing home	1.15	0.53–2.51	1.22	0.65–2.29	1.04	0.56–1.92
Walking ability indoor						
Independent	1.0	ref	1.0	ref	1.0	Ref
Dependent	2.32	0.99–5.38	1.46	0.70–3.07	1.27	0.60–2.69
Type of fracture						
Cervical	1.0	ref	1.0	ref	1.0	ref
Trochanteric	1.05	0.44–2.47	0.68	0.34–1.38	0.98	0.53–1.80
Pulmonary disease	1.49	0.60–3.67	**2.19**	1.10–4.39*	**2.60**	1.33–5.03*
Cerebrovascular disease	1.16	0.56–2.38	1.19	0.62–2.01	1.44	0.83–2.47
Cardiovascular disease	0.98	0.51–1.91	1.30	0.77–2.20	1.57	0.98–2.53
Diabetes	1.58	0.73–3.41	0.85	0.44–1.66	1.74	0.96–3.16
Cancer (current)	2.52	0.97–6.58	**4.23**	1.92–9.33*	**6.57**	2.83–15.29*
Depression, but not dementia	2.38	0.82–6.90	1.47	0.68–3.22	1.33	0.65–2.70
Dementia, but not depression	**4.05**	1.52–10.79*	**2.87**	1.38–5.98*	**2.55**	1.30–4.99*
Depression and dementia	**4.67**	1.75–12.51*	**3.61**	1.71–7.60*	**4.53**	2.24–9.14*

*CI* Confidence interval, *OR* Odds ratio

^*^Statistically significant *p* < 0.05

## Discussion

The main finding in our study was that DDwD were significantly associated with increased 12-, 24- and 36-month mortality risk following HF. In addition, patients with dementia, without DDs, also had increased mortality risk at all three follow ups.

The mortality risk is the highest the first year after hip fracture, which is also supported in by Katsoulis et al.’s [3]. Factors that contribute most to the increased mortality risk the first year are co-morbidities and numerous postoperative complications, such as infections, cardiac- and pulmonary complications [9]. The mortality risk will persist to be high even on a long-term, were the underlying excess risk remains more unclear [3]. DDwD increases short- and long-term mortality risks [33, 34]. Our findings are supported by Bellelli et al.’s [19], report of an increased 12-month mortality risk following HF among patients with DDwD, but their sample was small and from a rehabilitation unit, and thus not representative of all people sustaining HFs.

DDs are significantly more common among patients with dementia than among cognitively intact individuals [12]. DDs in older people are often underdiagnosed, undertreated, or untreated [35] possibly due to their complexity and similarity to other cognitive and psychiatric diseases. DDs may be a risk factor, prodromal symptom, or a consequence of dementia [36]. Older people have different pathophysiology and clinical presentation of DDs, compared to younger patients [37]. Further, several factors, such as biological- and psychosocial mechanisms, can contribute and be the cause of DDs among older people [38, 39], requiring a focus on addressing underlying causes.

Findings from younger populations with DDs may not be applicable to older populations, in which drug treatment alone has limited effects [40], especially among those with dementia [41, 42]. Combined psychosocial/drug interventions may yield better outcomes for patients with dementia [43–45]. The assessment and treatment of underlying risk factors (e.g. malnutrition and drug-side effects) is also important. Thus, the identification of alternative treatments or prevention of DDs in this population is important.

DDs influence functional outcome negatively after HF [46–48], which may be contributed by difficulties in goal-setting, planning, and rehabilitation-initiation [49]. Patients with dementia alone, and combined with DDs, are provided less rehabilitation, which can partly be influenced by the attitudes of the clinical staff [50]. However, patients with dementia benefit similarly, to those without dementia, from rehabilitation in a specialized geriatric ward after HF [51].

The identification of DDs in older people after HF surgery enables early intervention and minimize negative impacts on recovery and mortality. Clinical staff should provide early screening, especially for patients with dementia, to detect these disorders and provide adequate treatment, if indicated.

Limitations of this study include the use of old data from two RCTs and the operative treatment has changed since these studies were performed [52, 53]. However, the post-HF mortality rate has not changed in the past 25 years [2]. Other confounders, such as time-to-surgery [54], time to standing up after surgery [55] and socioeconomic status [56] could be potential factors that might contribute to increased mortality risk, but data were not available in the present study. A study strength derives from the same comprehensive assessment using validated scales, inclusion criteria, and methods in both RCTs. In addition, the same consultant geriatrician made all DDs and dementia diagnoses using the DSM-IV TR criteria, reducing the misclassification risk. For these reasons, the merging of data from the two RCTs to acquire a larger sample was possible.

## Conclusion

This study showed that patients with baseline DDwD have increased mortality risks at 12, 24, and 36 months after HF. Routinely assessments after HF for cognitive- and depressive disorders could identify patients at risk for increased mortality, and enable early interventions.

## Data Availability

The datasets used and/or analysed during the current study are available from the corresponding author on reasonable request.

## Abbreviations

ADL: Activity of daily living
CIs: Confidence intervals
DDs: Depressive disorders
DDwD: Dementia with depressive disorders
DSM-IV TR: Diagnostic and Statistical Manual of Mental Disorders, 4^th^ Edition
GDS-15: Geriatric Depression Scale
HF: Hip fracture
MMSE: Mini Mental state examination
OBS scale: The Organic Brain Syndrome scale
OR: Odds ratio
PGCMS: Philadelphia Geriatric Center Morale Scale
RCT: Randomized controlled trial

## References

[1] Abrahamsen B (2009). Excess mortality following hip fracture: a systematic epidemiological review. Osteoporos Int.

[2] Meyer AC (2021). Trends in Hip Fracture Incidence, Recurrence, and Survival by Education and Comorbidity: A Swedish Register-based Study. Epidemiology.

[3] Katsoulis M (2017). Excess mortality after hip fracture in elderly persons from Europe and the USA: the CHANCES project. J Intern Med.

[4] Haentjens P (2010). Meta-analysis: excess mortality after hip fracture among older women and men. Ann Intern Med.

[5] Smith T (2014). Pre-operative indicators for mortality following hip fracture surgery: a systematic review and meta-analysis. Age Ageing.

[6] Söderqvist A (2009). Prediction of Mortality in Elderly Patients with Hip Fractures: A Two-Year Prospective Study of 1,944 Patients. Gerontology.

[7] Baker NL, et al. Hip fracture risk and subsequent mortality among Alzheimer’s disease patients in the United Kingdom, 1988–2007. Age Ageing. 2011;40(1):49–54. 10.1093/ageing/afq146 21087990

[8] Karlsson Å (2020). Effects of Geriatric Interdisciplinary Home Rehabilitation on Independence in Activities of Daily Living in Older People With Hip Fracture: A Randomized Controlled Trial. Arch Phys Med Rehabil.

[9] Berggren M (2016). Co-morbidities, complications and causes of death among people with femoral neck fracture - a three-year follow-up study. BMC Geriatr.

[10] Hou M (2021). The effects of dementia on the prognosis and mortality of hip fracture surgery: a systematic review and meta-analysis. Aging Clin Exp Res.

[11] Sjöberg L (2017). Prevalence of depression: Comparisons of different depression definitions in population-based samples of older adults. J Affect Disord.

[12] Snowden MB (2015). Longitudinal Association of Dementia and Depression. Am J Geriatr Psychiatry.

[13] Pan CC (2018). Risk of hip fractures in patients with depressive disorders: A nationwide, population-based, retrospective, cohort study. PLoS ONE.

[14] Kim SY (2019). Depression and incident hip fracture: A longitudinal follow-up study using a national sample cohort. Medicine (Baltimore).

[15] Kvelde T (2013). Depressive Symptomatology as a Risk Factor for Falls in Older People: Systematic Review and Meta-Analysis. J Am Geriatr Soc.

[16] Read JR (2017). Multimorbidity and depression: A systematic review and meta-analysis. J Affect Disord.

[17] Guerini F (2010). Depressive symptoms and one year mortality among elderly patients discharged from a rehabilitation ward after orthopaedic surgery of the lower limbs. Behav Neurol.

[18] Liu Y, Wang Z, Xiao W (2018). Risk factors for mortality in elderly patients with hip fractures: a meta-analysis of 18 studies. Aging Clin Exp Res.

[19] Bellelli G (2008). Depressive symptoms combined with dementia affect 12-months survival in elderly patients after rehabilitation post-hip fracture surgery. Int J Geriatr Psychiatry.

[20] Stenvall M (2007). A multidisciplinary, multifactorial intervention program reduces postoperative falls and injuries after femoral neck fracture. Osteoporos Int.

[21] Karlsson Å (2016). Effects of Geriatric Interdisciplinary Home Rehabilitation on Walking Ability and Length of Hospital Stay After Hip Fracture: A Randomized Controlled Trial. J Am Med Dir Assoc.

[22] Hasselgren-Nyberg L, Omgren M, Nyberg L, Gustafsson Y (1992). Den svenska verionen av Physiotherapy Clinical Outcome Variables. Nordisk Fysioterapi.

[23] Sonn U (1996). Longitudinal studies of dependence in daily life activities among elderly persons. Scand J Rehabil Med Suppl.

[24] Sheikh JI, Yesavage JA (1986). Geriatric Depression Scale (GDS): Recent evidence and development of a shorter version. Clin Gerontol.

[25] Björkelund KB (2006). The Organic Brain Syndrome (OBS) scale: a systematic review. Int J Geriatr Psychiatry.

[26] Almeida OP, Almeida SA (1999). Short versions of the geriatric depression scale: a study of their validity for the diagnosis of a major depressive episode according to ICD-10 and DSM-IV. Int J Geriatr Psychiatry.

[27] Conradsson M (2013). Usefulness of the Geriatric Depression Scale 15-item version among very old people with and without cognitive impairment. Aging Ment Health.

[28] Folstein MF, Folstein SE, McHugh PR. “Mini-mental state”. A practical method for grading the cognitive state of patients for the clinician. J Psychiatr Res. 1975;12(3):189–98. 10.1016/0022-3956(75)90026-6 1202204

[29] Lawton MP. Lawton’s PGC Morale Scale. 2003. Available from: https://www.abramsoncenter.org/media/1198/lawtons-pgc-moral-scale.pdf. Cited 2020 1005.

[30] Niklasson J (2015). Psychometric properties and feasibility of the Swedish version of the Philadelphia Geriatric Center Morale Scale. Qual Life Res.

[31] Schober P, Boer C, Schwarte LA (2018). Correlation Coefficients: Appropriate Use and Interpretation. Anesth Analg.

[32] Bursac Z (2008). Purposeful selection of variables in logistic regression. Source Code Biol Med.

[33] Petersen JD (2017). Major Depressive Symptoms Increase 3-Year Mortality Rate in Patients with Mild Dementia. Int J Alzheimers Dis.

[34] Perna L (2019). Incident depression and mortality among people with different types of dementia: results from a longitudinal cohort study. Soc Psychiatry Psychiatr Epidemiol.

[35] Unützer J (2002). Diagnosis and treatment of older adults with depression in primary care. Biol Psychiatry.

[36] Aziz R, Steffens DC (2013). What are the causes of late-life depression?. Psychiatr Clin North Am.

[37] Schaakxs R (2017). Age-related variability in the presentation of symptoms of major depressive disorder. Psychol Med.

[38] Korten NCM (2012). Early and late onset depression in young and middle aged adults: Differential symptomatology, characteristics and risk factors?. J Affect Disord.

[39] Wetherell JL (2009). Older adults are less accurate than younger adults at identifying symptoms of anxiety and depression. J Nerv Ment Dis.

[40] Wilkinson P, Izmeth Z (2012). Continuation and maintenance treatments for depression in older people. Cochrane Database Syst Rev.

[41] Banerjee S (2011). Sertraline or mirtazapine for depression in dementia (HTA-SADD): a randomised, multicentre, double-blind, placebo-controlled trial. The Lancet.

[42] Weintraub D (2010). Sertraline for the Treatment of Depression in Alzheimer Disease: Week-24 Outcomes. Am J Geriatr Psychiatry.

[43] Areán PA (2010). Problem-solving therapy and supportive therapy in older adults with major depression and executive dysfunction. Am J Psychiatry.

[44] Gustavson KA (2016). Problem-Solving Therapy Reduces Suicidal Ideation In Depressed Older Adults with Executive Dysfunction. Am J Geriatr Psychiatry.

[45] Alexopoulos GS (2019). Mechanisms and treatment of late-life depression. Transl Psychiatry.

[46] Rathbun AM (2018). Persistence of depressive symptoms and gait speed recovery in older adults after hip fracture. Int J Geriatr Psychiatry.

[47] DubljaninRaspopović E (2014). Do depressive symptoms on hospital admission impact early functional outcome in elderly patients with hip fracture?. Psychogeriatrics.

[48] Morghen S (2011). Moderate to severe depressive symptoms and rehabilitation outcome in older adults with hip fracture. Int J Geriatr Psychiatry.

[49] Alexopoulos GS (2008). Anterior cingulate dysfunction in geriatric depression. Int J Geriatr Psychiatry.

[50] Bellelli G (2007). Does cognitive performance affect physical therapy regimen after hip fracture surgery?. Aging Clin Exp Res.

[51] Stenvall M (2012). A multidisciplinary intervention program improved the outcome after hip fracture for people with dementia–subgroup analyses of a randomized controlled trial. Arch Gerontol Geriatr.

[52] Sundkvist J (2021). Epidemiology, classification, treatment, and mortality of adult femoral neck and basicervical fractures: an observational study of 40,049 fractures from the Swedish Fracture Register. J Orthop Surg Res.

[53] Wolf O (2022). Increased mortality after intramedullary nailing of trochanteric fractures: a comparison of sliding hip screws with nails in 19,935 patients. Acta Orthop.

[54] Leer-Salvesen S (2019). Does time from fracture to surgery affect mortality and intraoperative medical complications for hip fracture patients? An observational study of 73 557 patients reported to the Norwegian Hip Fracture Register. Bone Joint J.

[55] Kristensen PK (2016). Are process performance measures associated with clinical outcomes among patients with hip fractures? A population-based cohort study. Int J Qual Health Care.

[56] Kristensen PK (2017). Socioeconomic inequality in clinical outcome among hip fracture patients: a nationwide cohort study. Osteoporos Int.

